# Prognostic significance of L-type amino acid transporter 1 expression in resectable stage I–III nonsmall cell lung cancer

**DOI:** 10.1038/sj.bjc.6604235

**Published:** 2008-02-05

**Authors:** K Kaira, N Oriuchi, H Imai, K Shimizu, N Yanagitani, N Sunaga, T Hisada, S Tanaka, T Ishizuka, Y Kanai, H Endou, T Nakajima, M Mori

**Affiliations:** 1 Department of Medicine and Molecular Science, Gunma University Graduate School of Medicine, Showa-machi, Maebashi, Gunma 371-8511, Japan; 2 Department of Diagnostic Radiology and Nuclear medicine, Gunma University Graduate School of Medicine, Showa-machi, Maebashi, Gunma 371-8511, Japan; 3 Department of Thoracic and Visceral Organ Surgery, Gunma University Graduate School of Medicine, Showa-machi, Maebashi, Gunma 371-8511, Japan; 4 Department of General Surgical Science, Gunma University Graduate School of Medicine, Showa-machi, Maebashi, Gunma 371-8511, Japan; 5 Division of Bio-system Pharmacology, Graduate School of Medicine, Osaka University, Suita, Osaka 565-087, Japan; 6 Department of Pharmacology and Toxicology, Kyorin University School of Medicine, Shinkawa, Mitaka, Tokyo 181-8611, Japan; 7 Department of Tumour Pathology, Gunma University Graduate School of Medicine, Showa-machi, Maebashi, Gunma 371-8511, Japan

**Keywords:** LAT1, nonsmall cell lung cancer, amino acid transporter, prognostic factor, Ki-67

## Abstract

The clinical significance of L-type amino acid transporter 1 (LAT1) expression remains unclear, whereas many experimental studies have demonstrated that LAT1 is associated with the proliferation of cancer cells. The purpose of this study was to evaluate the prognostic value of LAT1 in patients with nonsmall cell lung cancer (NSCLC). A total of 321 consecutive patients with completely resected pathologic stage I–III NSCLC were retrospectively reviewed. Expression of LAT1 and proliferative activity, as determined by the Ki-67 labelling index, was also evaluated immunohistochemically and correlated with the prognosis of patients who underwent complete resection of the tumour. Expression of LAT1 was positive in 163 patients (51%) (29% of adenocaricnoma (58 of 200 patients), 91% of squamous cell carcinoma (91 of 100 patients), and 67% of large cell carcinoma (14 of 21 patients)). The 5-year survival rate of LAT1-positive patients (51.8%) was significantly worse than that of LAT1-negative patients (87.8%; *P*<0.001). L-type amino acid transporter 1 expression was significantly associated with lymph node metastasis and disease stage. Multivariate analysis confirmed that positive expression of LAT1 was an independent factor for predicting a poor prognosis. There was a significant correlation between LAT1 expression and Ki-67 labelling index. LAT1 expression is a promising pathological factor to predict the prognosis in patients with resectable stage I–III NSCLC.

Nonsmall cell lung cancer (NSCLC) is the leading cause of cancer death and has a poor prognosis (Shottenfeld, 1996). To improve the prognosis of patients, clinical markers that may predict the prognosis and response to the specific therapy should be established. Tumour staging and performance status have been consistently shown to be the most powerful prognostic tool for survival rates of NSCLC patients (Brundage *et al*, 2002). The prognostic factors such as ‘N’ factor, tumour size, sex, and vessel invasion have been shown to determine overall outcome in patients with surgically resected NSCLC (Brundage *et al*, 2002). Recently, excision repair cross-complementation group 1 has been described to be a new promising marker for drug response and overall survival in NSCLC patients (Olaussen *et al*, 2006). However, there has been no established clinical marker, which correlates with the response to the treatment and the prognosis in patients with NSCLC (Clinical practice guidelines for the treatment of unresectable nonsmall cell lung cancer, 1997).

Amino acid transporters are essential for growth and proliferation in normal and transformed cells (Christensen, 1990; McGivan and Pastor-Anglada, 1994). Among amino acid transporters, system L is a Na^+^-independent large and neutral amino acid transport agency (Oxender and Christensen, 1962; Christensen, 1990). L-type amino acid transporter 1 (LAT1) is one of the L-type amino acid transporters, and transports large neutral amino acids such as leucine, isoleucine, valine, phenylalanine, tyrosine, tryptophan, methionine and histidine (Oxender and Christensen, 1962; Kanai *et al*, 1998; Yanagida *et al*, 2001). L-type amino acid transporter 1 requires covalent association with the heavy chain of 4F2 cell surface antigen (4F2hc) for its functional expression in plasma membrane (Oxender and Christensen, 1962). Previous studies have shown LAT1 to be highly expressed in proliferating tissues, many tumour cell lines (T24 bladder carcinoma cells, RERF-LC-MA lung small-cell carcinoma cells, and HeLa uterine cervical carcinoma cells) and primary human tumours (Yanagida *et al*, 2001). It has been reported that LAT1 expression is closely related to tumour cell growth of liver metastases in a rat model (Ohkame *et al*, 2001). Recent studies demonstrated the overexpression of LAT1 in pulmonary adenocarcinoma and oesophageal carcinoma (Kobayashi *et al*, 2005; Nakanishi *et al*, 2006). High LAT1 immunostaining was described to predict a poor prognosis in patients with astrocytic brain tumours (Nawashiro *et al*, 2006). The RNA interference to downregulate LAT1 has been described to impair the proliferation of human oral cancer cells (Kim *et al*, 2006). Although many experimental studies have clearly indicated that LAT1 is associated with cancerous or proliferative cells, the clinical significance of LAT1 expression in NSCLC remains unclear (Yanagida *et al*, 2001; Nakanishi *et al*, 2006). Since there is no clinical study on LAT1 expression in NSCLC, the correlation between LAT1 expression and prognosis is also unknown. In this study, therefore, the pathological findings including LAT1 expression was assessed in the resected tissue specimen and correlated with clinical feature and outcome. In addition, LAT1 expression was correlated with the proliferative activity of the tumour as assessed by the Ki-67 labelling index.

## MATERIALS AND METHODS

### Patients

We analysed 361 consecutive patients with NSCLC who underwent resection either by lobectomy or pneumonectomy with mediastinal lymph node dissection at Gunma University Hospital between June 1998 and May 2004. Nineteen patients who received induction of chemotherapy or radiation therapy and one patient who suffered from an operation-related death were excluded. Specimens of 20 patients were not available. A total of 321 patients (196 men, 125 women) were evaluated. The study protocol was approved by the institutional review board.

The age of the patients ranged from 39 to 84 years, and the mean age at the time of surgery was 67 years. The tumour specimens were histologically classified according to the criteria of the World Health Organization. Postsurgical pathologic stage was determined by the current tumour-node-metastasis classification (Mountain, 1997). Histologically, 200 patients had adenocarcinoma, 100 had squamous cell carcinoma (SQC), and 21 had large cell carcinoma (LCC). Of the total patients, 241, 28 and 52 had stage I, II and III tumours, respectively. As postoperative adjuvant therapies, platinum-based chemotherapy, radiation, and oral administration of tegafur (a fluorouracil derivative drug) were administered to 27, 14, and 35 patients, respectively. Intraoperative therapy was not performed on any patient. The postoperative clinical course was assessed by analysing outpatient medical records and by marking telephone inquiries. The day of surgery was considered the starting day for counting postoperative survival. The follow-up duration ranged from 6 to 86 months (median, 48 months).

### Immunohistochemical staining with LAT1 and Ki-67

#### LAT1

L-type amino acid transporter 1 expression was determined by immunohistochemical staining with an affinity-purified polyclonal rabbit anti-human LAT1 antibody (Yanagida *et al*, 2001). An oligopeptide corresponding to amino acid residues 497–507 of human LAT1 (CQKLMQVVPQET) was synthesized. The N-terminal cysteine residue was introduced for conjugation with keyhole limpet hemocyanine. Antipeptide antibody was produced as described elsewhere (Chairoungdua *et al*, 1999). For immunohistochemical analysis, antiserum was affinity-purified as described previously (Chairoungdua *et al*, 1999).

Immunohistochemical staining was performed on paraffin sections using a polymer peroxidase method (Envision+/HRP; Dako Cytomation CO., Ltd., Denmark). Briefly, deparaffinised and rehydrated sections were treated with 0.3% hydrogen peroxide in methanol for 30 min to block endogenous peroxidase activity. To expose antigens, sections were autoclaved in 10 mmol l^−1^ sodium citrate buffer (pH 6.0) for 5 min, and cooled for 30 min. After rinsing in 0.05 M tris-buffered saline containing 0.1% tween 20, the sections were incubated with affinity-purified anti-LAT1 antibody (1.2 mg ml^−1^; 1 : 3200) overnight at 4°C. Thereafter, they were incubated with Envision (+) rabbit peroxidase (Dako, Carpinteria, CA, USA) for 30 min. The peroxidase reaction was performed using 0.02% 3,3′-diaminobenzidine tetrahydrochloride and 0.01% hydrogen peroxide in 0.05 M tris–HCl buffer, pH 7.4. Finally, nuclear counterstaining was performed with Mayer's hematoxylin. For negative control, incubation step with the primary antibody was omitted. The specificity of immunoreactions using the anti-LAT1 antibody was established in previous studies (Matsuo *et al*, 2000; Nawashiro *et al*, 2006).

LAT1 expression was considered positive only if distinct membrane staining was present. Staining intensity was scored as follows: (1) <10% of tumour area stained; (2) 11–25% stained; (3) 26–50% stained; and (4) ⩾51% stained. The tumours in which stained tumour cells made up more than 10% of the tumour were graded as positive. According to this scoring protocol, two investigators from the authors, without prior knowledge of the clinical data, independently graded the staining intensity in all cases. To test the intraobserver variability, each section was reassessed by the same investigators after the first assessment had been completed. The time interval between the first and second assessments was at least 4 weeks. The interobserver variability was also determined by comparing the values of the first measurements of two investigators.

#### Ki-67

The detailed protocol for immunostaining was published elsewhere (Buck *et al*, 2001). Briefly, formalin-fixed and paraffin-embedded sections of resected specimens were dewaxed, rehydrated, trypsinized, and boiled in 0.01 mol/l. citrate buffer for 30 min. For immunostaining, the monoclonal mouse antibody MIB-1 (Dako, Denmark), specific for human nuclear antigen Ki-67, was used in a 1 : 40 dilution. The sections were lightly counterstained with hematoxylin. As a positive control for proliferating cells, sections of tonsil were used.

A highly cellular area of the immunostained sections was evaluated. All epithelial cells with nuclear staining of any intensity were defined as positive. Approximately 1000 nuclei were counted on each slide. Proliferative activity was assessed as the percentage of MIB-1-stained nuclei in the sample. Ki-67 expression was defined as low if less than 25% of tumour cells showed staining in nuclei in a tumour section. This definition was used according to the commonly used cutoff values ranging from 20 to 40% in NSCLC and other human cancers in the current literature (Martin *et al*, 2004) and also based on the examination of our staining data. Sections were evaluated by two investigators separately and in case of discrepancies both would evaluate the slide simultaneously and would agree in their final assessment. Neither investigators had knowledge of patient outcome.

### Statistical analysis

The *χ*^2^-test and Fisher's exact test was used to examine the association of two categorical variables. Correlations between LAT1 expression and Ki-67 labelling index were analysed by using the nonparametric Spearman's rank test.

Survival time was determined as the time from tumour resection to death from any cause. For survivors, survival times were censored on the last date that patients were known to be alive. The Kaplan–Meier method was used to estimate survival as a function of time, and survival differences were analysed by the log-rank test. Multivariate analyses were performed using stepwise Cox proportional hazards model to identify independent prognostic factors. A *P*-value less than 0.05 was considered indicative of statistical significance. Statistical analysis was performed using StatView J-4.5 for Macintosh.

## RESULTS

### Expression of LAT1 in NSCLC

**Figure 1:**
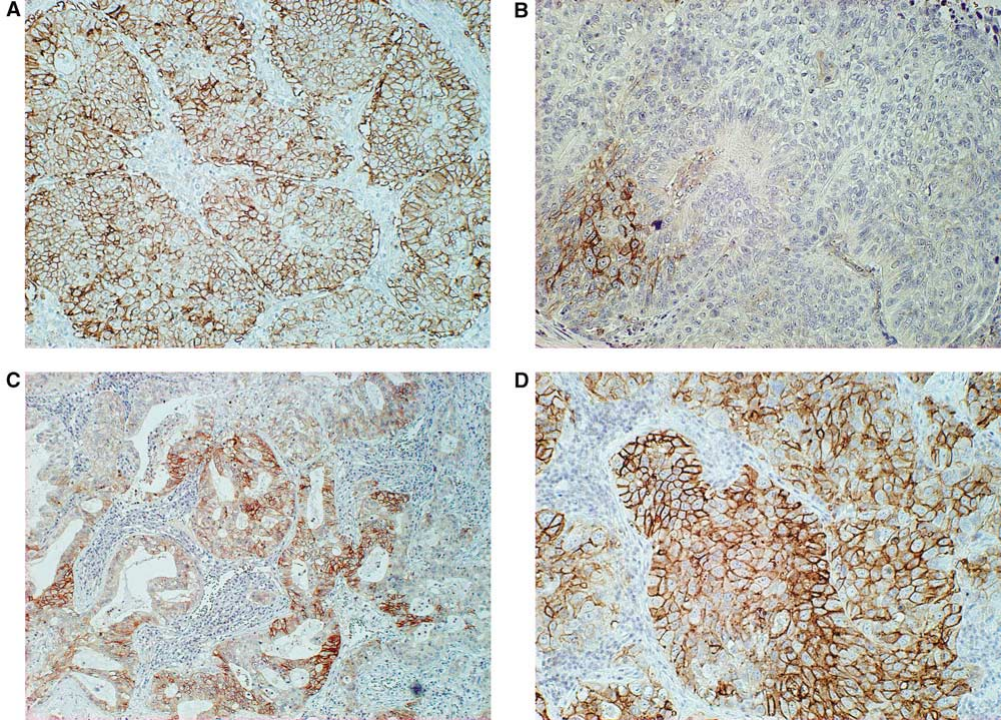
Immunohistochemical analysis of LAT1 in NSCLC. (**A**) Positive staining of LAT1 expression in squamous cell carcinoma. The score of LAT1 immunostaining was grade 4 and its immunostaining pattern was membranous. (**B**) LAT1 expression in squamous cell carcinoma with staining score of grade 2. (**C**) LAT1 expression in adenocarcinoma with staining score of grade 3. (**D**) LAT1 expression in large cell carcinoma with staining score of grade 4.

L-type amino acid transporter 1 immunostaining was detected in carcinoma cells in tumour tissues and localised predominantly on their plasma membrane (Figure 1). In the present study, no expression of LAT1 protein was observed in any normal epithelial cells of the lung, including bronchial epithelial and alveolar cells. All positive cells revealed strong membranous LAT1 immunostaining. It is worthy to note that the staining intensity of the cell membrane and the percentage of positive cells were considerably greater in SQC and LCC than AC. The cytoplasmic staining was rarely evident. Expression of LAT1 was positive in 51% (163 of 321 patients) (29% of AC (58 of 200 patients), 91% of SQC (91 of 100 patients), and 67% of LCC (14 of 21 patients)). The incidence of a positive LAT1 expression was significantly different between AC and SQC (*P*<0.001) and between AC and non-AC (*P*<0.001). The average score of the LAT1 expression was 2.0±1.2 on a scale of 1–4. The LAT1 score was 1.5±0.8 in AC, 3.1±1.0 in SQC and 2.5±1.3 in LCC. There was a significant difference in the LAT1 score between AC and SQC (*P*<0.001) and between AC and LCC (*P*<0.001).

Expression of LAT1 according to patient's characteristics is listed in Table 1. In NSCLC, LAT1 expression was significantly associated with gender, tumour size, lymph node metastasis, disease stage, lymphatic permeation, vascular permeation, and pleural involvement. In AC, LAT1 expression was significantly associated with gender, lymph node metastasis, disease stage, lymphatic permeation, vascular permeation, and pleural involvement. In SQC, LAT1 expression was significantly associated with lymph node metastasis.

### LAT1 expression and postoperative survival

**Figure 2:**
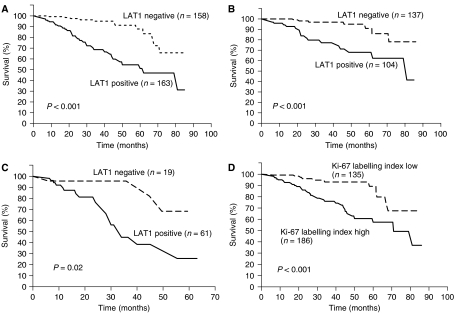
Postoperative survival of patients with completely resected pathologic stage I–III non-small cell lung cancer. Comparison of postoperative survival rates were based on LAT1 expression and Ki-67 labelling index. The survival rate of LAT1-positive patients was significantly worse than that of LAT1-negative patients with stage I–III NSCLC (*P*<0.001) (**A**). The survival rate of LAT1-positive patients was also associated with an unfavourable prognosis in stage I (*P*<0.001) (**B**) and stage II+III NSCLC (*P*=0.02) (**C**). The survival rate of patients with high Ki-67 labelling index was significantly worse than that with low Ki-67 labelling index (*P*<0.001) (**D**).

Postoperative survival according to LAT1 status is listed in Table 2. For all patients, the 5-year survival rates of the LAT1-positive and LAT1-negative patients were 51.8 and 87.8%, respectively, demonstrating a significantly poor prognosis for LAT1-positive patients (*P*<0.001, Figure 2A, B and C). Postoperative survival was also analysed by age, gender, and pathologic stage. For pathologic stage I disease, the 5-year survival rates of LAT1-positive and LAT1-negative patients were 67.9 and 90.9%, respectively, demonstrating a significantly poor prognosis for the LAT1-positive patients (*P*<0.001, Table 2). For pathologic stage II and III disease, the 5-year survival rates of LAT1-positive and LAT1-negative patients were 25.6 and 68.3%, respectively, demonstrating a significantly poor prognosis for the LAT1-positive patients (*P*=0.021, Table 2). A significant difference in the prognosis between the LAT1-positive and LAT1-negative patients was also demonstrated for age and gender (Table 2).

### LAT1 and Ki-67 expression

The Ki-67 labelling index averaged 35±24% (median, 32%) and ranged from 5 to 92% in NSCLC. The Ki-67 labelling index was 23±20% (median, 18%) in AC, 54±17% (median, 56%) in SQC and 56±21% (median, 63%) in LCC. The Ki-67 labelling index was significantly different between AC and SQC (*P*<0.001) and between AC and LCC (*P*<0.001).

**Figure 3:**
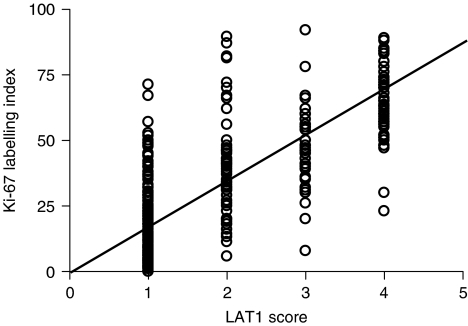
Correlation of LAT1 expression with Ki-67 labelling index (*γ*=0.772, *P*<0.001).

Significant correlation was found between LAT1 expression and Ki-67 labelling index (Spearman's rank correlation coefficient *γ*=0.772, *P*<0.001) in NSCLC (AC (*γ*=0.5817, *P*<0.001), SQC (*γ*=0.6917, *P*<0.001), and (LCC (*γ*=0.7947, *P*<0.001)) (Figure 3). Moreover, Kaplan–Meier analysis demonstrated that 5-year survival rate of patients with high Ki-67 labelling index tumours significantly decreased in comparison with those with low Ki-67 labelling index tumours (57.5 *vs* 89.2%; *P*<0.001) (Figure 2D).

### Multivariate analysis of prognostic factors

Multivariate analysis confirmed that positive expression of LAT1 was an independent and significant factor to predict a poor prognosis. Pathologic stage was also a significant prognostic factor (Table 3).

## DISCUSSION

The present study evaluated the clinical significance of LAT1 expression in NSCLC. The results of the study clearly demonstrated that the expression of LAT1, and the pathologic disease stage, was a significant independent factor to predict a poor prognosis in patients with completely resected NSCLC. Moreover, our study has shown that LAT1 expression was significantly associated with lymph node metastasis and disease stage.

It is widely known that amino acid transport systems play an important role in the regulation of cellular proliferation, whereas the details of its function to promote tumour cell proliferation have not been clarified (Kanai *et al*, 1998). Full-length LAT1 was first isolated and characterised in 1998 (Kanai *et al*, 1998). LAT1 is widely expressed in primary human cancers and several cancer cell lines, where it has been shown to play essential roles in growth and survival (Fuchs and Bode, 2006). While it is currently unclear why LAT1 is coveted by transformed cell, Fuchs *et al* hypothesize that LAT1 provides the essential amino acids that act as signal to enhance growth of cancer cells via mammalian target-of-rapamycin (mTOR)-stimulated translation (Fuchs and Bode, 2006). Likewise, mTOR regulates amino acid transporter gene expression and trafficking to the plasma membrane in response to the growth signal (Fuchs and Bode, 2006). Moreover, overexpression of LAT1 was described to be associated with metastasis *in vivo* (Ohkame *et al*, 2001; Tamai *et al*, 2001). When colon cancer RCN-9 cells were injected into the spleen of rats, the size of the resultant metastatic liver tumours was directly correlated to LAT1 expression (Ohkame *et al*, 2001; Tamai *et al*, 2001). Thus, inhibition of LAT1 function could be a potential therapeutic strategy for many types of cancer (Kanai and Endou, 2001; Fuchs and Bode, 2006). Whereas, LAT1 has been shown to mediate the uptake of a number of amino acid-related compounds such as L-dopa, triiodothyronine, thyroxin, and the anti-cancer agent melphalan (L-phenylalanine mustard, L-PAM, Alkeran, L-Sarcolysin) (Uchino *et al*, 2002). LAT1 appears to have opposing roles for the growth of cancer cell and also the suppression of the growth. It is a potential target *per se* because it supplies tumour cells with essential amino acids necessary for protein synthesis and cell growth. Conversely, its upregulation in a variety of cancers might be exploited for anti-tumour drugs like melphalan.

L-type amino acid transporter 1 protein overexpression in bronchioalveolar carcinoma is shown to associate with the Ki-67 labelling index, indicating an upregulation of metabolic activity (Nakanishi *et al*, 2006). Our result revealed that Ki-67 labelling index is significantly correlated with LAT1 expression in NSCLC. Ki-67 labelling index in SQC and LCC was significantly higher than that in AC. A meta-analysis indicated that the expression of Ki-67 is a factor of poor prognosis for survival in NSCLC (Martin *et al*, 2004). The present study revealed that high Ki-67 labelling index is associated with an unfavourable prognosis in patients with completely resected NSCLC.

We examined LAT1 expression immunohistochemically and found that LAT1 expression in SQC and LCC was significantly higher than that in AC. Since the LAT1 expression was significantly correlated with Ki-67 labelling index, the incidence of LAT1 expression in NSCLC may be associated with tumour cell proliferation. However, the reason why the incidence of LAT1 expression was different among the histopathologic subtypes is not known and remained to be elucidated.

Several clinical investigations demonstrated the increased uptake of radiolabelled amino acids in human neoplasms (Inoue *et al*, 2001; Oriuchi *et al*, 2006; Kaira *et al*, 2007b). We have developed L-[3-^18^F]-*α*–methyltyrosine (FMT) as a tracer for amino acid transport using positron emission tomography (PET) imaging (Tomiyoshi *et al*, 1997), and investigated the clinical utility of FMT in several tumours including brain tumour, lung cancer, head and neck cancer, and lymphoma (Oriuchi *et al*, 2006). FMT is transported via L-type amino acid transporter, which is specific to cancer cells (Kim *et al*, 2002; Oriuchi *et al*, 2006; Kaira *et al*, 2007b). Recently, we reported a significant correlation between FMT uptake and LAT1 expression in NSCLC (Kaira *et al*, 2007a).

In conclusion, positive expression of LAT1 is a significant factor to predict poor prognosis, and it may be an important clinical marker of therapy for NSCLC. LAT1 expression was significantly correlated with tumour cell proliferation. Inhibiting LAT1 function may cause a cessation of the growth of tumour and provide new and effective therapeutic target of NSCLC in the future.

## Figures and Tables

**Table 1:** Association between LAT1 expression and the clinicopathological features

	**All^a^**			**Adenocarcinoma**			**Squamous cell carcinoma**		
		**LAT1 positive**			**LAT1 positive**			**LAT1 positive**	
**Parameter**	**No.**	**No. (%)**	***P*-value**	**No.**	**No. (%)**	***P*-value**	**No.**	**No. (%)**	***P*-value**
Total	321	163 (51)		200	58 (29)		100	91 (91)	
									
*Age*									
⩽65 years	105	45 (43)	0.186	77	20 (26)	0.280	20	18 (90)	0.74
>65 years	216	118 (55)		123	38 (31)		80	73 (91)	
									
*Gender*									
Male	205	131 (64)	<0.001^b^	94	34 (36)	0.025	92	83 (90)	0.456
Female	116	32 (28)		106	24 (23)		8	8 (100)	
									
*Tumour size (mm)*									
⩽30	159	84 (53)	0.022	108	38 (35)	0.738	47	42 (89)	0.423
>30	124	81 (65)		54	21 (39)		53	49 (92)	
									
*Lymph node metastasis*									
Positive	56	39 (70)	<0.001	30	16 (52)	<0.001	18	18 (100)	<0.001
Negative	265	24 (9)		170	14 (8)		82	0 (0)	
									
*Disease stage (p-stage)*									
I	241	104 (43)	<0.001	164	38 (23)	<0.001	69	61 (88)	0.59
II+III	80	61 (76)		36	21 (58)		15	14 (93)	
									
*Lymphatic permeation*									
Positive	126	87 (69)	<0.001	65	31 (48)	<0.001	51	46 (90)	0.526
Negative	194	77 (40)		135	27 (20)		49	45 (92)	
									
*Vascular invasion*									
Positive	94	64 (68)	<0.001	45	19 (42)	0.022	40	36 (90)	0.74
Negative	227	100 (44)		155	39 (25)		60	55 (92)	
									
*Pleural involvement*									
Positive	103	66 (64)	<0.001	55	25 (45)	<0.001	34	30 (88)	0.855
Negative	219	98 (45)		145	33 (23)		66	61 (92)	

LAT1=L-type amino acid transporter 1.

a Large cell carcinoma was included. ^b^Statistically significant.

**Table 2:** Five-year survival according to LAT1 expression

	**Five-year survival rate (%)^a^**		
**Variable**	**LAT1 positive**	**LAT1 negative**	***P*-value^b^**
All patients	51.8	87.8	<0.001
			
*Age*			
⩽ 65 years	60.0	90.0	0.008
>65 years	46.9	83.1	<0.001
			
*Gender*			
Male	47.7	79.3	0.006
Female	63.9	98.2	<0.001
			
*Pathologic stage*			
I	67.9	90.9	<0.001
II+III	25.6	68.3	0.021

LAT1=L-type amino acid transporter 1.

a Kaplan–Meier analysis. ^b^Log-rank test.

**Table 3:** Multivariate analysis of the prognostic factors

**Prognostic factor**	**Hazard ratio**	**95% confidence interval**	***P*-value**
Age (⩽65 years/>65 years)	0.597	0.299–1.194	0.1447
Gender (male/female)	1.398	0.658–2.972	0.3839
Histology (adenocarcinoma/squamous cell carcinoma)	1.228	0.535–2.816	0.6277
Disease stage (I/II+III)	3.544	1.788–7.026	0.0003
LAT1 (positive/negative)	3.243	1.356–7.755	0.0082
			
*Postoperative adjuvant therapy*			
Cisplatin-based intravenous chemotherapy (no/yes)	0.835	0.287–2.431	0.7405
Radiation therapy (no/yes)	1.135	0.316–4.078	0.8456
Oral administration of tegafur (no/yes)	1.320	0.562–3.101	0.5236
Ki-67 labeling index (low/high)	0.667	0.271–1.643	0.3789

LAT1=L-type amino acid transporter 1.

Note: Hazard ratios, 95% confidence intervals, and two-side *P*-values were obtained from the Cox proportional hazards models.
